# Ethnicity and socioeconomic status are related to dietary patterns at age 5 in the Amsterdam born children and their development (ABCD) cohort

**DOI:** 10.1186/s12889-017-5014-0

**Published:** 2018-01-08

**Authors:** Viyan Rashid, Marielle F. Engberink, Manon van Eijsden, Mary Nicolaou, Louise H. Dekker, Arnoud P. Verhoeff, Peter J. M. Weijs

**Affiliations:** 1 Department of Nutrition and Dietetics, Faculty of Sports and Nutrition, Amsterdam University of Applied Sciences, Dr. Meurerlaan 8, 1067 SM Amsterdam, The Netherlands; 2 Department of Epidemiology, Health Promotion and Health Care Innovation, Public Health Service Amsterdam, Amsterdam, The Netherlands; 3 Department of Public Health, Academic Medical Center, University of Amsterdam, Amsterdam Public Health Institute, The Netherlands; 4 Department of Sociology, University of Amsterdam, Amsterdam, The Netherlands; 5 Nutrition and Dietetics, Department of Internal Medicine, VU University Medical Center, Amsterdam, The Netherlands

**Keywords:** Dietary patterns, PCA, Children, Preschool children, Ethnicity, Socioeconomic status, Overweight

## Abstract

**Background:**

Health inequalities are already present at young age and tend to vary with ethnicity and socioeconomic status (SES). Diet is a major determinant of overweight, and studying dietary patterns as a whole in relation to overweight rather than single nutrients or foods has been suggested. We derived dietary patterns at age 5 and determined whether ethnicity and SES were both related to these dietary patterns.

**Methods:**

We analysed 2769 validated Food Frequency Questionnaires filled in by mothers of children (5.7 ± 0.5y) in the Amsterdam Born Children and their Development (ABCD) cohort. Food items were reduced to 41 food groups. Energy adjusted intake per food group (g/d) was used to derive dietary patterns using Principal Component Analysis and children were given a pattern score for each dietary pattern. We defined 5 ethnic groups (Dutch, Surinamese, Turkish, Moroccan, other ethnicities) and 3 SES groups (low, middle, high, based on maternal education). Multivariate ANOVA, with adjustment for age, gender and maternal age, was used to test potential associations between ethnicity or SES and dietary pattern scores. Post-hoc analyses with Bonferroni adjustment were used to examine differences between groups.

**Results:**

Principal Component Analysis identified 4 dietary patterns: a snacking, full-fat, meat and healthy dietary pattern, explaining 21% of the variation in dietary intake. Ethnicity was related to the dietary pattern scores (*p* < 0.01): non-Dutch children scored high on snacking and healthy pattern, whereas Turkish children scored high on full-fat and Surinamese children on the meat pattern. SES was related to the snacking, full-fat and meat patterns (*p* < 0.01): low SES children scored high on the snacking and meat pattern and low on the full-fat pattern.

**Conclusions:**

This study indicates that both ethnicity and SES are relevant for dietary patterns at age 5 and may enable more specific nutrition education to specific ethnic and low socioeconomic status target groups.

**Electronic supplementary material:**

The online version of this article (10.1186/s12889-017-5014-0) contains supplementary material, which is available to authorized users.

## Background

Health inequalities, such as the prevalence of overweight, are already present at a young age and tend to vary on the basis of ethnicity and socioeconomic (SES) status [1–3]. Diet is a major determinant of overweight [4–6], and studying dietary patterns as a whole in relation to overweight rather than single nutrients or foods has been suggested [7–9].

Dietary patterns are population specific and influenced by sociocultural factors and food availability [10, 11]. In recent decades, European populations have become increasingly ethnically diverse and ethnic minority groups are often disproportionate in lower SES groups [12]. The predominant ethnic minority groups, i.e. Turkish, Arabs (North African and Middle Eastern), Berbers and Black Africans (Afro-Caribbean and others by descent), form approximately 3% of the total European population, with the largest numbers in Western European countries [13]. Non-native groups have less often completed higher education than native borns [14] which makes observation of SES differences also of interest.

Socioeconomic differences in dietary patterns have been described in adults. In children, SES differences in dietary patterns has been observed in several studies including 4 prospective birth cohorts in 3 countries in Europe, i.e. The Avon Longitudinal Study of Parents and Children (ALSPAC) cohort, the EDEN mother-child cohort, the Norwegian Mother and Child Cohort Study and the Southampton Women’s Cohort Survey [15–23]. Data on ethnic differences in dietary patterns among children is limited [24]. To our knowledge, only the ALSPAC cohort identified an association between ethnicity dividing the study population into white and non-white ethnicity [18, 19]. However, the diversity of ethnic groups in Western Europe is more pronounced and we expect to observe differences in dietary intake between ethnic groups [25–27]. Exploring the potential ethnic diversity as well as socioeconomic differences in dietary patterns in children may provide new and more specific insight for public healthcare professionals to identify groups with poor dietary habits.

Therefore, the aim of the present study was to derive dietary patterns at age 5 in the multi-ethnic Amsterdam Born Children and their Development (ABCD) cohort and to examine potential associations with either or both ethnicity and SES.

## Methods

### Study design and study population

**Fig. 1:**
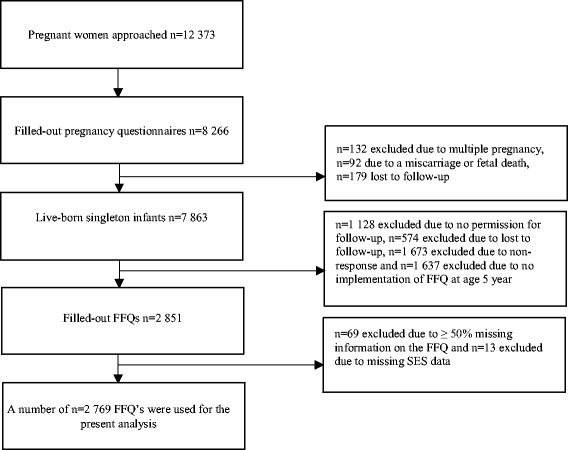
Flowchart of the inclusion into the present analysis (*n* = 2769)

Data were used from the ABCD study, a large ongoing community-based birth cohort (http://www.abcd-study.nl/). The cohort study design has been described previously [28]. Figure 1 shows the study procedure and inclusion in the current analysis. In brief, between January 2003 and March 2004, all pregnant women living in Amsterdam were invited to participate in the ABCD study by their obstetric care provider at their first prenatal care visit. Of the 12,373 women approached, 8266 women filled out a pregnancy questionnaire that covered socio-demographic characteristics, obstetric history, family history and lifestyle, which was available in Dutch, English, Turkish and the Arabic language. When the children turned 5 years of age, 4488 received a self-administered Food Frequency Questionnaire (FFQ) by post and a number of 2851 mothers returned the FFQ. Based on a data clearance protocol set by TNO Food (Zeist, The Netherlands), children were excluded from analysis with more than 50% missing per page or per cluster of food items (*n* = 69). Finally, 13 children were excluded as years of education of the mother was not available in the pregnancy questionnaire, resulting in 2769 children included in the present analysis.

Efforts to enhance participation among all women and children, regardless of ethnicity and education were done by using translated questionnaires and information leaflets. Also, women from ethnic minority groups who did not respond within a month were approached by phone by trained students who explained the study in the women’s preferred language. Attrition in follow-up number was largely attributable to untraceable changes in address or migration. This study was approved by the institutional review committee of the Academic Medical Center, and the Registration Committee of Amsterdam. All of the participants gave written informed consent for themselves and their children. The present study was conducted according to the guidelines laid down in the Declaration of Helsinki.

### Dietary assessment

A validated 71-item FFQ, developed by TNO Food (Zeist, The Netherlands) was used [29]. Per food item, consumption frequency, portion size and the type of product consumed over the last 4 weeks was reported by the mother of the child. Frequency options were “never”, “less than once a week”, “once a week”, “2–3 times a week”, “4–5 times a week”, and “6–7 times a week”. Food items were assessed in units (e.g. a piece of fruit and a slice of bread) and in household units (e.g. a glass and a tablespoon). Intake of items such as breakfast cereals, vegetables or pasta were asked in standard tablespoons which could give a reliable idea of the actual eaten portion size for children. Based on the data clearance protocol developed by TNO Food, the returned FFQs were scanned and the data were checked for inconsistencies or extreme values. Impossible values were defined as portion sizes larger than the maximum portion size consumed in the Dutch Food Consumption Survey and were imputed by the mean. For example a maximum of 6 tablespoons of cooked vegetables (180 g) per day was substituted when a higher amount was filled in. Frequencies and portion sizes were converted into weights (g/day) of product consumed and intake of energy was calculated using the Dutch Food Composition Database (NEVO) 2010 [30]. Each food item in the questionnaire was linked with one or more foods from the Dutch Food Composition Database. In total, a number of 308 different NEVO codes were used for analysis. After calculation of the scanned FFQs, inconsistencies in energy intake for those children with the 5% highest and 5% lowest intake of energy were checked with the original FFQ. When the FFQ was filled in correctly, FFQ’s of these children were not excluded as we expect that these high or low energy intakes might reflect a realistic intake.

### Assessment of ethnicity and socioeconomic status

Data on ethnicity and SES was collected via the pregnancy questionnaire, filled out by the mother during the baseline measurements of the ABCD study. Five ethnic categories were formed: Dutch, Surinamese, Turkish, Moroccan and other ethnicities (mainly non-western origin). We excluded the Surinamese South Asians because of specific body composition and cardiometabolic risk [31]. Ethnicity was based on the country of birth of the pregnant woman and her mother including both first-generation women (born outside the Netherlands) and second generation women (born in the Netherlands but whose mother was born in another country). When the pregnant woman or her mother were born in a country defined as ‘none of the given options’: the participant’s self-registered ethnic origin was used [32].

The pregnant women’s education after primary school was defined in years and considered as a proxy for SES. Low SES was defined as a maximum of 5 years post-primary education, middle SES as 6–10 years and high SES was defined as more than 10 years of post-primary education [33].

### Assessment of dietary patterns

Principal Component Analyses (PCA) with varimax rotation was used to derive dietary patterns. Food items, including different type of products, were reduced to 41 food groups, based on nutritional value and culinary use. The list with food groups and its type of products can be found in Additional file 1. Products such as ginger cake and raisins are often given to children as a healthy alternative for biscuits or candy and were therefore assigned to the food group “healthy snacks”. Because we were interested in the effect of dietary quality independent of its energy content, we adjusted total energy intake using the nutrient residual method [34, 35]. Standardized energy adjusted intake (g/d) of the 41 food groups were used in the PCA analysis. The number of components (dietary patterns) retained was based on the scree plot [see Additional file 2], eigenvalues >1 and the interpretability of the dietary patterns [36, 37]. Food groups with component loadings ≥0.3 were considered important for interpretability of the dietary patterns. A larger absolute factor loading indicates a higher positive or negative correlation between the food group and a given dietary pattern. The patterns were named after the nature of the food groups with the highest component loadings within each pattern.

Individuals were given a pattern score for each pattern as a sum of the 41 standardized food group intakes, each weighted according to their factor loading. Positive pattern scores indicate higher consumption of food groups in that pattern.

### Statistical analysis

Statistical analyses were performed in SPSS version 22 for windows. Population characteristics were described in percentages or means with standard deviations (SD), shown for the total population and by ethnicity. Univariate and multivariate ANOVA was used to determine whether ethnicity and/or SES were related to dietary patterns with the individual pattern score of each dietary pattern used as continuous dependent variable and ethnicity or SES used as independent variables (Model 1; crude). The association with ethnicity was additionally adjusted for SES (dummy) and the association with SES was additionally adjusted for ethnicity (dummy) (Model 2). In the fully adjusted model (Model 3) the association with ethnicity was adjusted for child’s age (y), gender, maternal age (y) and SES (dummy) and the analysis with SES was adjusted for age (y), gender, maternal age (y) and ethnicity (dummy). Mean ± SE pattern scores were shown for each of the dietary patterns by ethnic and SES group separately. Post-hoc analyses with Bonferroni adjustment was used to examine differences between groups. Additionally, we tested for interaction by SES in the association between ethnicity and dietary pattern scores. *P* < 0.01 was considered significant.

## Results

### Population characteristics

Characteristics of the study population, divided by ethnicity are shown in Table 1. Mean age of the study population was 5.7 ± 0.5 years and 51% of the population was boy. The percentage of children from Dutch origin was 82.4%, followed by Surinamese (4.2%), Moroccan (4.1%), Turkish (2.2%) and other ethnicities (7.1%). The majority of children (53.3%) belonged to the high SES, 35.4% to middle SES and 11.3% to low SES group.

**Table 1:** Population characteristics in the ABCD cohort by ethnicity (*n* = 2769)

Population characteristics	Total population (*n* = 2769)	Dutch (*n* = 2283, 82.4%)	Surinamese (*n* = 116, 4.2%)	Moroccan (*n* = 112, 4.1%)	Turkish (*n* = 61, 2.2%)	other ethnicities (*n* = 197, 7.1%)
Age, in year (Mean, SD)	5.7, 0.5	5.7, 0.5	5.8, 0.5	6.0, 0.6	5.9, 0.5	5.7, 0.5
Boy, *n* (%)	1415 (51.1)	1166 (51.1)	58 (50.0)	64 (57.1)	34 (55.7)	93 (47.2)
Socioeconomic status, *n* (%)						
Low	313 (11.3)	145 (6.4)	38 (32.8)	48 (42.9)	33 (54.1)	49 (24.9)
Middle	980 (35.4)	759 (33.2)	57 (49.1)	55 (49.1)	25 (41.0)	84 (42.6)
High	1476 (53.3)	1379 (60.4)	21 (18.1)	9.(8.0)	3 (4.9)	64 (32.5)
Maternal age (Mean, SD)	32.3, 4.3	32.8, 3.8	30.6, 5.8	27.9, 4.9	27.1, 6.0	31.5, 4.8

Ethnicity was based on the country of birth of the pregnant woman and her mother including both first-generation women and second generation women. SES was based on maternal educational: low SES (≤ 6y), middle SES (6-10y) and high SES (≥10y) post-primary education.

### Dietary patterns

PCA identified 4 dietary patterns in this cohort explaining 20.8% of the variation of dietary intake, according to the Rotated Sums of Squared Loadings. In Table 2, an overview of the component loadings from ≥0.3 is shown per dietary pattern. The snacking pattern was mainly characterized by high intakes of savoury snacks and refined breakfast products and low intakes of whole-grain breakfast products. The full-fat pattern was characterized by high intakes of full-fat spreads and pasta dishes and low intakes of low-fat spreads. The meat pattern was characterised by high intakes of low- and high-fat meat, sauces and refined grain products for warm meals. Finally the healthy pattern was characterised by high intakes on the food groups water and tea, vegetables, fish and fruits.

**Table 2:** Component loadings (≥0.3) of the 41 food groups per dietary pattern

	Dietary patterns			
	Snacking	Full-fat	Meat	Healthy
Explained variance (%)	7.1	4.6	4.6	4.4
Savory snacks	0.47	-	-	-
Refined breakfast products	0.45	–	–	–
Ice cream	0.42	–	–	–
Sauces	0.41	–	0.35	–
Choclate and candy	0.38	–	–	–
Fruit drink	0.31	–	–	–
Full-fat dairy	0.30	–	–	–
Low-fat spreads	−0.38	−0.55	–	–
Sandwich toppings (sweet)	−0.38	–	–	−0.33
Whole grain breakfast products	−0.74	–	–	–
Tomato sauce for pasta	–	0.61	–	–
Full-fat spreads	–	0.48	−0.30	–
Refined grain products warm meal	–	0.46	0.34	–
Full-fat cheese	–	0.37	–	–
Low-fat cheese	–	−0.35	–	–
Low-fat meat	–	–	0.44	–
High-fat meat	–	–	0.39	–
Healthy meals	–	–	0.31	–
Boiled potatoes	–	–	0.30	–
Unhealthy meals	–	–	−0.32	–
Peanut butter	–	–	−0.34	–
Water and tea	–	–	–	0.48
Vegetables	–	–	–	0.47
Fish	–	–	–	0.46
Fruits	–	–	–	0.38
Whole grain products warm meal	–	–	–	0.36
Nuts	–	–	–	0.31
Pulses	–	–	–	0.30
Artificially sweeted sodas	–	–	–	–
Biscuits and pastries	–	–	–	–
Egg	–	–	–	–
Fried potato products	–	–	–	–
Fruit drink concentrate	–	–	–	–
Granola bars	–	–	–	–
Healthy snacks	–	–	–	–
Low-fat dairy	–	–	–	–
Meat alternatives and soy products	–	–	–	–
Medium-fat dairy	–	–	–	–
Processed meats	–	–	–	–
Sugar	–	–	–	–
Sugar sweeted sodas	–	–	–	–
a. Rotation converged in 12 iterations				

Component loadings (≥0.3) were considered important for interpretability of the dietary patterns. A larger factor loading indicates a higher positive or negative correlation between the food group and dietary pattern

### Ethnicity and dietary patterns

Ethnicity was significantly related to dietary pattern scores (*p* < 0.01, Table 3). Post-hoc analyses showed that Dutch children had significantly lower (−0.171 ± 0.019, *p* < 0.01) snacking scores compared to the other ethnic groups, whereas Turkish children had significantly higher (1.363 ± 0.118, *p* < 0.01) snacking scores. After adjustment for SES the associations were less pronounced (−0.124 ± 0.019 for Dutch, 0.998 ± 0.117 for Turkish), but still significant for most groups. Further adjustment for age, gender and maternal age did not change the results (Table 3). With respect to the full-fat pattern, Turkish children and children from other ethnicities had higher pattern scores compared to Moroccan children (0.283 ± 0.128 and 0.167 ± 0.071 versus −0.247 ± 0.094, *p* < 0.01), whereas Surinamese children scored higher on the meat pattern (0.589 ± 0.092) compared to the other ethnic groups (p < 0.01). Adjustment for SES did somewhat diminish the associations, but not the level of significance (Table 3). Further adjustment for other confounding factors yielded similar results (Table 3). The healthy pattern was most pronounced within the groups of Turkish and Moroccan children (0.660 ± 0.092 for Moroccan and 0.602 ± 0.125 for Turkish, *p* < 0.01). Adjustment for SES and other factors did not change the results.

**Table 3:** Mean dietary pattern scores by ethnicity in the ABCD cohort (*n* = 2769)

Dietary pattern	Dutch		Surinamese		Turkish		Moroccan		other ethnicities		*P* value
	Mean	SE	Mean	SE	Mean	SE	Mean	SE	Mean	SE	ANOVA
Snacking											
Model 1: Crude	−0.171	0.019^a^	0.797	0.086^b,d^	1.363	0.118^a^	0.819	0.087^b,d^	0.626	0.066^b,d^	<0.01
Model 2: SES	−0.124	0.019^a^	0.576	0.084^b^	0.998	0.117^b,e,f^	0.516	0.087^b,d^	0.491	0.064^b,d^	<0.01
Model 3: Fully adjusted	−0.122	0.019^a^	0.567	0.084^b^	0.987	0.119^b,e,f^	0.505	0.089^b,d^	0.485	0.064^b,d^	<0.01
Full-fat											
Model 1: Crude	−0.003	0.021	−0.127	0.093	0.283	0.128^e^	−0.247	0.094^d,f^	0.167	0.071^e^	<0.01
Model 2: SES	−0.019	0.021	−0.054	0.094	0.409	0.131^e^	−0.146	0.097^d^	0.212	0.072	<0.01
Model 3: Fully adjusted	−0.021	0.021^d^	−0.054	0.094	0.433	0.132^b,e^	−0.136	0.099^d^	0.217	0.072	<0.01
Meat											
Model 1: Crude	−0.020	0.021^c^	0.589	0.092^a^	−0.088	0.127^c^	−0.001	0.094^c^	−0.088	0.071^c^	<0.01
Model 2: SES	0.002	0.021^c^	0.484	0.093^a^	−0.253	0.130^c^	−0.143	0.096^c^	−0.152	0.071^c^	<0.01
Model 3: Fully adjusted	0.007	0.021^c^	0.469	0.093^a^	−0.297	0.131^c^	−0.182	0.098^c^	−0.157	0.071^c^	<0.01
Healthy											
Model 1: Crude	−0.085	0.020^d,e,f^	0.023	0.091^d,e,f^	0.602	0.125^b,c^	0.660	0.092^b,c^	0.415	0.070^b,c^	<0.01
Model 2: SES	−0.085	0.021^d,e,f^	0.023	0.092^d,e,f^	0.597	0.129^b,c^	0.659	0.095^b,c^	0.415	0.071^b,c^	<0.01
Model 3: Fully adjusted	−0.089	0.021^d,e,f^	0.033	0.092^d,e,f^	0.645	0.130^b,c^	0.703	0.097^b,c^	0.414	0.070^b,c^	<0.01

Ethnicity was based on the country of birth of the pregnant woman and her mother including both first-generation women and second generation women.

Mean, SE pattern score per dietary pattern by ethnicity.

Mean pattern scores for the total group was set to 0.000 based on PCA method.

Model 1: unadjusted.

Model 2: adjusted for SES.

Model 3: adjusted for SES, age, gender and maternal age.

Sign (*P* < 0.01) is based on ANOVA and Post-hoc Bonferroni.

^a^sign with all groups

^b^sign with Dutch

^c^sign with Surinamese

^d^sign with Turkish

^e^sign with Moroccan

^f^sign with other ethnicities

### Socioeconomic status and dietary patterns

SES was significantly related to snacking, full-fat and meat dietary pattern scores (*p* < 0.01, Table 4). Post-hoc analyses showed that low SES children had significantly higher snacking pattern scores (0.864 ± 0.052) compared to middle (0.171 ± 0.030) and high SES groups (−0.297 ± 0.024, *p* < 0.01). After adjustment for ethnicity the associations were less pronounced (0.590 ± 0.054 for low SES, 0.137 ± 0.029 for middle SES and −0.216 ± 0.024 for high SES), but still significant. Further adjustment for age, gender and maternal age did not change the results. The full-fat pattern was most pronounced within the group of high SES children (0.055 ± 0.026, *p* < 0.01). The meat pattern was most pronounced in low SES children (0.229 ± 0.056, *p* < 0.01). After adjustment for ethnicity, associations were more pronounced (Table 4). Further adjustment for age, gender and maternal age did not change the results (Table 4). SES was significantly related to the healthy pattern in the crude model (*p* < 0.01, Table 4) showing low SES children had higher healthy pattern scores (0.217 ± 0.056) compared to middle (0.004 ± 0.032) and high SES children (−0.049 ± 0.026). After adjustments in either model 2 or 3, SES was no longer significantly associated with the healthy pattern.

**Table 4:** Mean dietary pattern scores by socioeconomic status in the ABCD cohort (*n* = 2769)

Dietary pattern	Low SES		Middle SES		High SES		*P* value
	Mean	SE	Mean	SE	Mean	SE	ANOVA
Snacking							
Model 1: Crude	0.864	0.052^a^	0.171	0.030^a^	−0.297	0.024^a^	<0.01
Model 2: Ethnicity	0.590	0.054^a^	0.137	0.029^a^	−0.216	0.024^a^	<0.01
Model 3: Fully adjusted	0.591	0.054^a^	0.134	0.029^a^	−0.214	0.024^a^	<0.01
Full-fat							
Model 1: Crude	−0.179	0.056^b^	−0.026	0.032	0.055	0.026^c^	<0.01
Model 2: Ethnicity	−0.217	0.060^b^	−0.028	0.032	0.065	0.027^c^	<0.01
Model 3: Fully adjusted	−0.213	0.060^b^	−0.025	0.032	0.061	0.027^c^	<0.01
Meat							
Model 1: Crude	0.229^b^	0.056	0.098	0.032^b^	−0.114	0.026^a^	<0.01
Model 2: Ethnicity	0.242	0.060^b^	0.096	0.032^b^	−0.115	0.026^a^	<0.01
Model 3: Fully adjusted	0.231	0.060^b^	0.093	0.032^b^	−0.111	0.026^a^	<0.01
Healthy							
Model 1: Crude	0.217	0.056^a^	0.004	0.032^c^	−0.049	0.026^c^	<0.01
Model 2: Ethnicity	0.025	0.059	−0.019	0.031	0.008	0.026	0.716
Model 3: Fully adjusted	0.043	0.059	−0.019	0.031	0.003	0.026	0.618

SES was based on maternal educational: low SES (<6y), middle SES (6-10y) and high SES (>10y) post-primary education.

Mean, SE pattern scores per dietary pattern by socioeconomic group.

Mean pattern scores for the total group was set to 0.000, based on PCA method.

Model 1: unadjusted.

Model 2: adjusted for ethnicity.

Model 3: adjusted for ethnicity, age, gender and maternal age.

Sign (*P* < 0.01) is based on ANOVA and Post-hoc Bonferroni.

^a^sign with all groups

^b^sign with high SES group

^c^sign with low SES group

### Ethnicity, socioeconomic status and dietary patterns

**Fig. 2:**
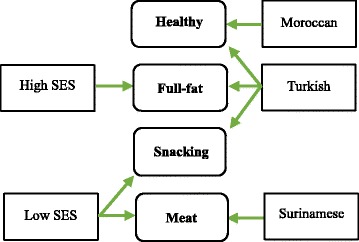
The main positive significant associations between ethnicity, SES and dietary patterns (*n* = 2769)

The main positive significant associations between ethnicity, SES and dietary patterns in the fully adjusted model are shown in Fig. 2. We tested for interaction between SES and ethnicity in relation to pattern scores and found a borderline significant interaction for the full-fat (*p* = 0.018) and meat pattern (*p* = 0.017), whereas no interaction was present for the snacking (*p* = 0.324) and healthy (*p* = 0.260) pattern. Profile plots showed that both ethnicity and SES were independently related to dietary patterns [See Additional file 3].

## Discussion

We have identified four dietary patterns in the large multi-ethnic ABCD cohort, consisting of 2769 children. Already at age 5, both ethnicity and SES were independently related to dietary patterns. Non-Dutch had high snacking and healthy pattern scores, whereas Turkish children scored higher on full-fat and Surinamese children scored higher on meat pattern scores. Low SES children had high snacking, meat and low full-fat pattern scores. Both ethnicity and SES seem to contribute independently to the differences in dietary patterns.

### Interpretation and comparison with previous studies

Results of a systematic review including 14 publications utilizing PCA in 1–8 year old native children in mainly European countries [15] showed that most studies identified between two and six dietary patterns, with the majority of studies identifying a healthy, unhealthy/processed/snacking, and local/traditional pattern [5, 15, 22, 38]. Among the cohorts that evaluated the diets of children aged 3–5 years, a healthy and unhealthy pattern were most often identified [15, 17, 21, 38–42] with similar dietary patterns as the healthy and snacking pattern, which were observed in the present analysis. Our full-fat pattern shows similarities with the varied traditional Norwegian pattern, found by Oellingrath in 9–10 year old Norwegian children, which was characterized by high component loadings on full-fat cheese and full-fat spreads [5], food groups that also characterized the full-fat pattern in this study.

We have identified an association between ethnicity and dietary patterns. Up to now, data on the association between ethnicity and dietary patterns has been scarce. In ALSPAC a snacking pattern was related to white ethnicity at age 3 and 7 [17, 19] and a healthy pattern with non-white ethnicity at age 4 to 7 years [19]. We found both higher healthy and snacking pattern scores in non-Dutch groups. In the Netherlands, the consumption of fruit and vegetables is higher in 7–9 year old children from Turkish and Moroccan origin [43] and 9–10 year old children from non-western origin [44] than that of Dutch children. However also consumption of snacking items and soft drinks has been found to be higher in 5-6y old non-ethnic groups, mainly of Turkish origin [42] which is in line with findings in our study. Additionally, the present study showed a full-fat and a meat pattern. Surinamese children have higher meat pattern scores than children from other ethnic groups and Turkish children have higher full-fat pattern scores. It has been reported in Dutch National Food Survey’s that intake of fat and full-fat dairy products is high among groups from Turkish origin [25, 26].

Several studies have observed SES differences in dietary patterns in children [15–23] with maternal education being the most important variables [18, 21]. In four large prospective birth cohorts (ALSPAC, the EDEN mother-child cohort, the Norwegian Mother and Child Cohort Study and the Southampton Women’s Cohort Survey) healthier dietary patterns in young children (1-7y) were associated with higher maternal education [17, 19, 21, 23, 39]. We did not find significantly different healthy pattern scores between SES groups however low SES children had higher healthy pattern scores than middle and high SES groups. In the ALSPAC cohort, the junk pattern at age 4 and 7 was more likely when maternal education level was low [20]. In line with these findings, we found that low SES children have higher snacking pattern scores. Our high SES children had higher full-fat pattern scores (full-fat spreads, full-fat cheese and refined pasta dishes) and low meat pattern scores (low- and high fat meat, sauces and refined grain products for warm meals). We did not find other studies describing this full-fat dietary pattern in high SES children.

In the present study, the non-Dutch groups (Surinamese, Turkish, Moroccan and other ethnicity) came disproportionally from lower SES groups (Table 1). Although ethnicity and SES are strongly correlated, we showed that both ethnicity and SES explained differences in dietary pattern scores between groups at age 5y. This suggests that both ethnicity and SES seem to be a predictor for adherence to a specific dietary pattern.

### Methodological consideration

A problem common in studies using the PCA method is that the number of dietary patterns is based on scree plots, eigenvalues and the interpretability of the dietary patterns which is a limitation in objectivity [45]. The labelling of the identified patterns is subjective, which can be judged by the reader from the presented component loadings (Table 2). The 4 identified dietary patterns in this cohort explained 20.8% of the variation of dietary intake which is common in studies using the PCA method.

FFQs are considered an appropriate method for population-based evaluations of dietary patterns in childhood and are favoured in large-scale studies because they are less burdensome to participants and reduce post-collection processing of dietary data [27]. A possible limitation is that the FFQ was based on food commonly consumed by the Dutch population as determined by the Dutch Food Consumption Survey 1997–1998 [46]. However subanalysis showed that energy intake related to energy requirements (based on Schofield resting metabolism) was not different between Dutch and non-Dutch groups. It might be possible that some ethnic specific food items were not registered by the mother. The open question at the end of the FFQ gave the mother the opportunity to register consumed food items that were not literally asked in the 71-items. Based on methodological considerations (not all mothers filled-in this open question and there was the risk of double registration), we decided to not analyse these registered items. The FFQ was validated with the gold standard of doubly labelled water in a group of 4- to 6-year-old children, who did not exactly reflect the non-Dutch groups [29].

The present study had a response rate of 33% of the original cohort. Smaller numbers in ethnic groups is inherent to the ABCD study design but it is possible that some biases may have been introduced into the analyses, particularly as the nonresponders tended to come disproportionately from lower SES and ethnic minority groups that consumed more according to the snacking pattern. Response rates per ethnic and SES group were 53% for Dutch, 23% for Surinamese, 14% for Turkish, 15% for Moroccan, 9% for other ethnicities, 16% for low SES, 31% for middle SES and 47% for high SES. A nonresponse analysis determining the degree of selective response and selection bias between pregnancy and birth outcomes, indicated that selective non-response was present in the ABCD-study, but selection bias was acceptably low and did not influence the studied birth outcomes [47].

Strengths of this study includes the sample size of 2769 children in which dietary pattern analyses was performed. The present study is one of few that provides insight into dietary patterns in children in a multi-ethnic population.

### Implications for research and interventions

In this group of young children, we identified specific ethnic and SES groups that consumed more according to unfavourable dietary patterns. Dietary tracking, the maintenance of a dietary pattern over a certain time period, has been observed during childhood and from childhood to adolescence and unhealthy eating habits have been found to track into adolescence and adulthood.

Dietary habits are a major determinant of overweight [4, 5]. Non-native, especially children of Turkish origin, and low SES groups show higher adherence to the unfavourable snacking pattern and show disproportionally higher prevalence of overweight and obesity (7% for Dutch, 14% for Surinamese, 25% for Turkish, 23% for Moroccan, 17% for low SES, 12% for middle SES and 8% for high SES) at age 5 [48]. Future studies could analyse the explanatory factors in early childhood contributing to these (differences in) dietary choices and the possible relationships these dietary patterns may have with weight development and health inequalities in later childhood.

## Conclusion

This study indicates that both ethnicity and SES are relevant for dietary patterns at age 5 and may enable more specific nutrition education to specific ethnic and low SES target groups, in order to avoid overweight and other health inequalities.

## Additional files

Additional file 1: Food groups and their products, used in the PCA analysis in the ABCD cohort (*n*=2 769). (PDF 23 kb)

Additional file 2: Scree plot of the 41 components in the PCA in the ABCD cohort (n=2 769). (PDF 36 kb)

Additional file 3: Profile plots of the interaction between ethicity and SES per dietary pattern (*n*=2 769). (PDF 92 kb)

## Abbreviations

ABCD: Amsterdam Born Children and their Development
SES: socioeconomic status
ANOVA: Analysis of Variance
ALSPAC: The Avon Longitudinal Study of Parents and Children
FFQ: Food Frequency Questionnaire
PCA: Principal Component Analysis
