# Supplementary material for: Ethnicity and socioeconomic status are related to dietary patterns at age 5 in the Amsterdam born children and their development (ABCD) cohort
Source: BMC Public Health. 2018 Jan 8;18:115. doi: 10.1186/s12889-017-5014-0 (PMC5759294; doi:10.1186/s12889-017-5014-0)
Supplement: Supplementary file 1 — Food groups and their products, used in the PCA analysis in the ABCD cohort (n=2 769). (PDF 23 kb) [file 12889_2017_5014_MOESM1_ESM.pdf]

Additional file 1. Food groups and their products, used in the PCA analysis in the ABCD cohort (n=2 769).

Food group	Products assigned to the food group
**Fruits**	All sorts of fruits
**Vegetables**	All sorts or raw and boiled vegetables (excluding vegetables in the mashed potato stew)
**Refined breakfast products**	White/refined bread, rusks, crackers, crispbreads, Cornflakes, Cruesli, Rice Krispies, Frosties, Smacks, Honey Loops
**Whole grain breakfast products**	Brown- and whole grain bread, rusks, crackers, crispbreads and rye bread, muesli
**Low-fat spreads**	Low-fat margarine, Blue Band Good Start
**Full-fat spreads**	Butter, margarine
**Low-fat cheese**	Low-fat or light cheese (spreads) 20+ 30+
**Full-fat cheese**	Full-cream cheese (spreads) 48+, Brie 60+, goat cheese, including cheese added to hot meals
**Processed meats**	Ham, corned beef, liver products, salami, meatloaf, smoked dried beef
**Peanut butter**	Peanut butter
**Sandwich toppings (sweet)**	Chocolate spreads and sprinkles, jam, apple syrup, honey, fruit sprinkles, coconut slices, Schuddebuikjes
**Low-fat dairy**	Skimmed, low-fat or artificially sweetened vanilla, fruit or natural quark/yoghurt(drink)/Biogarde/(chocolate) milk, buttermilk
**Medium-fat dairy**	Semi-skimmed vanilla, fruit or natural quark/yoghurt(drink)/Biogarde/(chocolate) milk, Barley gruel
**Full-fat dairy**	Custard, milk pudding, Danoontje, full-cream vanilla, fruit or natural quark/yoghurt(drink)/Biogarde/(chocolate) milk, ready-made porridge from oats, semolina or rice
**Unhealthy meals**	Pizza, quiche, filled tortillas or taco's, poffertjes, pancackes
**Healthy meals**	Mashed potato stew with vegetables, nasi, bami, noodles
**Tomato sauce (for pasta)**	Tomato sauce in pasta dishes
**Refined grain products**	White rice, macaroni, spaghetti or other pasta
**Whole grain products warm meal**	Whole grain rice, macaroni, spaghetti or other pasta
**Fried potato products**	Rösti, fried potato or French fries
**Boiled potatoes**	Boiled or mashed potatoes
**Sauces**	Gravy, mayonaise or salad cream
**Pulses**	Brown, white beans and marrowfat peas etc.
**Fish**	All types of fish
**Eggs**	Boiled and baked eggs
**Low-fat meat**	Chicken breast or leg, chop, pork slices/steak/filet, stewing beef or steak, steak tartar, roast beef,
**High-fat meat**	Chicken burger/nuggets, minced meat, bacon, sausage, hamburger, schnitzel, frankfurters or cocktail sausages as a snack or on bread
**Meat alternatives and soy products**	Flavoured or natural soy milk(dessert). Vegetarian schnitzel/burger, tahoe/tofu, tempé, *Quorn* or *Valess.*
**Granola bars**	Granola bars, Sultana, Liga nutritional biscuits
**Biscuits and pastries**	Sweet pie, pastries, fruit flan, cakes and biscuits
**Ice cream**	Ice cream
**Chocolate and candy**	All types of choclate bars, chewing gum and sweets

**Healthy snacks**	Ginger cake, sponge cake, raisins, ice lolly, breadsticks or rice cakes
**Savory snacks**	Kroket, frikandel, satay, crisps, nibbits, prawn crackers, salty biscuits, sausage roll
**Nuts**	(coated) peanuts
**Sugar sweetened sodas**	Sugar containing soft drinks and squashes
**Artificially sweetened sodas**	Light and artificially sweetened soft drinks and squashes
**Fruit drink**	Apple juice, orange juice, Dubbeldrank and multi-fruit drink
**Fruit drink concentrate**	Fruit concentrate diksap, Roosvicee and Karvan Cevitam
**Water and tea**	(Mineral)water and tea without sugar
**Sugar**	Added sugar or calorie containing sweeteners in cereals, desserts or tea
