# Supplementary figures and images for: Ethnicity and socioeconomic status are related to dietary patterns at age 5 in the Amsterdam born children and their development (ABCD) cohort
Source: BMC Public Health. 2018 Jan 8;18:115. doi: 10.1186/s12889-017-5014-0 (PMC5759294; doi:10.1186/s12889-017-5014-0)

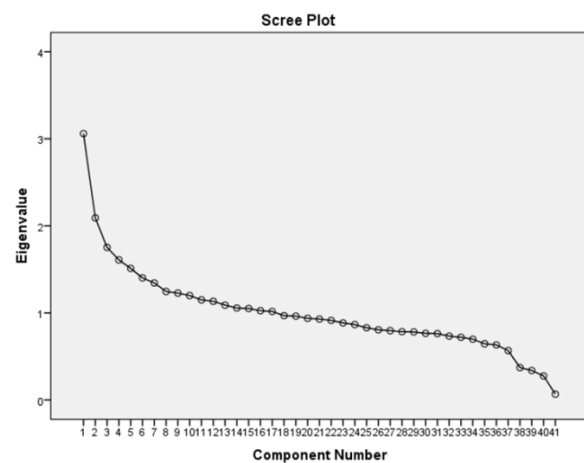

Additional file 2. Scree plot of the 41 components in the PCA in the ABCD cohort (n=2 769).

Supplement: Supplementary file 2 — Scree plot of the 41 components in the PCA in the ABCD cohort (n=2 769). (PDF 36 kb) [file 12889_2017_5014_MOESM2_ESM.pdf]
