# Supplementary material for: Ethnicity and socioeconomic status are related to dietary patterns at age 5 in the Amsterdam born children and their development (ABCD) cohort
Source: BMC Public Health. 2018 Jan 8;18:115. doi: 10.1186/s12889-017-5014-0 (PMC5759294; doi:10.1186/s12889-017-5014-0)
Supplement: Supplementary file 3 — Profile plots of the interaction between ethicity and SES per dietary pattern (n=2 769). (PDF 92 kb) [file 12889_2017_5014_MOESM3_ESM.pdf]

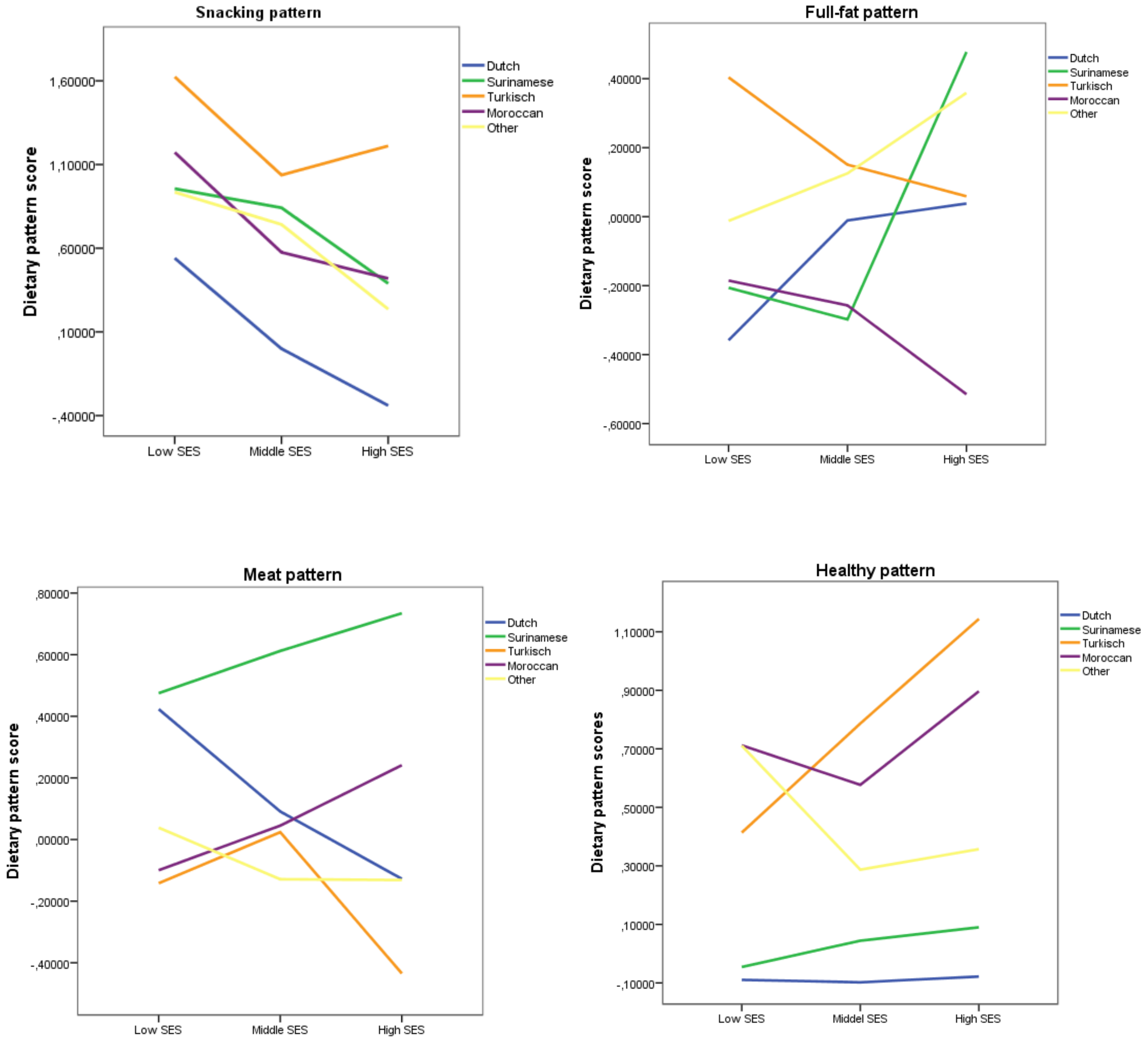

Additional file 3. Profile plots of the interaction between ethicity and SES per dietary pattern (n=2 769).
